# Wound Healing Promotion via Release of Therapeutic Metallic Ions from Phosphate Glass Fibers: An *In Vitro* and *Ex Vivo* Study

**DOI:** 10.1021/acsami.4c07035

**Published:** 2024-07-16

**Authors:** Agron Hoxha, Athanasios Nikolaou, Holly N. Wilkinson, Matthew J. Hardman, Jorge Gutierrez-Merino, Monica Felipe-Sotelo, Daniela Carta

**Affiliations:** † School of Chemistry and Chemical Engineering, University of Surrey, Guildford GU2 7XH, U.K.; ‡ School of Biosciences and Medicine, University of Surrey, Guildford GU2 7XH, U.K.; § Centre for Biomedicine, Hull York Medical School, University of Hull, Hull HU6 7RX, U.K.; ∥ Skin Research Centre, Hull York Medical School, University of York, York YO10 5DD, U.K.

**Keywords:** wound healing, phosphate-based glass fibers, antibacterial, soft tissue regeneration, controlled release

## Abstract

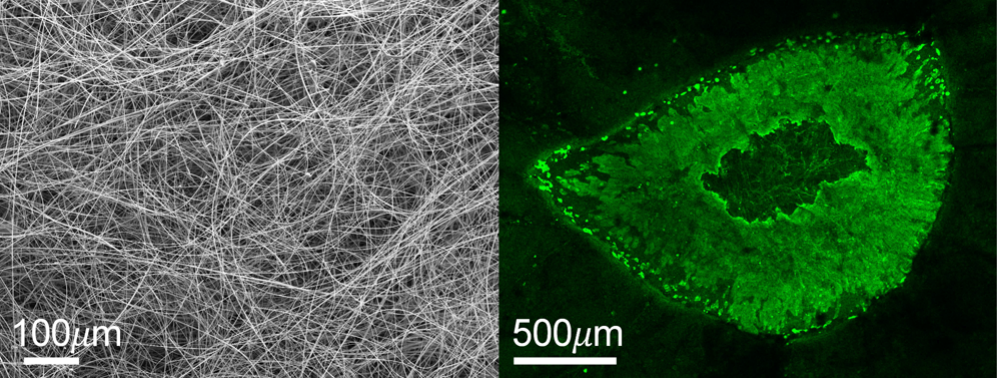

Biomaterials capable of promoting wound healing and preventing infections remain in great demand to address the global unmet need for the treatment of chronic wounds. Phosphate-based glasses (PG) have shown potential as bioresorbable materials capable of inducing tissue regeneration, while being replaced by regenerated tissue and releasing therapeutic species. In this work, phosphate-glass-based fibers (PGF) in the system P_2_O_5_–CaO–Na_2_O added with 1, 2, 4, 6, and 10 mol % of the therapeutic metallic ions (TMI) Ag^+^, Zn^2+^, and Fe^3+^ were manufactured via electrospinning of coacervate gels. Coacervation is a sustainable, cost-effective, water-based method to produce PG. All TMI are effective in promoting wound closure (re-epithelialization) in living human skin *ex vivo*, where the best-performing system is PGF containing Ag^+^. In particular, PGF with ≥4 mol % of Ag^+^ is capable of promoting 84% wound closure over 48 h. These results are confirmed by scratch test migration assays, with the PGF-Ag systems containing ≥6 mol % of Ag^+^, demonstrating significant wound closure enhancement (up to 72%) after 24 h. The PGF-Ag systems are also the most effective in terms of antibacterial activity against both the Gram-positive *Staphylococcus aureus* and the Gram-negative *Escherichia coli*. PGF doped with Zn^2+^ shows antibacterial activity only against *S. aureus* in the systems containing Zn^2+^ ≥ 10 mol %. In addition, PGF doped with Fe^3+^ rapidly accelerates *ex vivo* healing in patient chronic wound skin (>30% in 48 h), demonstrating the utility of doped PGF as a potential therapeutic strategy to treat chronic wounds.

## 1 Introduction

There is a large global unmet need for the treatment of non-healing chronic wounds (*e.g.*, venous ulcers, pressure ulcers, and diabetic foot ulcers). Delayed wound healing has a great impact on patients’ quality of life, resulting in pain, social isolation, extended hospital stay, and even death.^1^ Individuals with delayed wound healing pose a high risk of developing bacterial infections resistant to antibiotics, commonly from *Staphylococcus aureus* and *Escherichia coli*, which further hinders healing.^2^

The aim of this work is to address a clinical urgent unmet need for affordable, bioresorbable wound repair materials able to both stimulate healing and deliver sustained antibacterial activity (preventing/treating infection). Currently, tissue-engineered products that provide a scaffold for wound repair (matrices) include skin autograft (transfer of healthy patient’s own skin), skin allograft (donor’s skin), or xenograft (animal tissue). These matrices have severe limitations: pain, rejection, limited availability, variable immune responses, or transmission of diseases.^3^ The decellularization process often used to minimize inflammatory/immunogenic responses of biological matrices is expensive, delivers variable quality, and frequently leads to rapid degradation. Fully synthetic products overcome these limitations, avoiding the risk of disease transmission and offering control of the composition/morphology of the matrices. Synthetic wound matrices, often based on polymers (*e.g.*, polyglycolic/polylactic acid, polycaprolactone), can cause toxicity and inflammation due to the accumulation of crystalline/acidic degradation products.^4,5^ Natural polymers can also be used (*e.g.*, chitosan, alginate, and gelatin).^6^ However, synthetic and natural polymers can often have low biocompatibility, unpredictable solubility, poor mechanical properties and lack of cell-binding sites; for these reasons, they are frequently functionalized and/or combined with biological materials in composite matrices.^7^ Therefore, there is an urgent need to develop biomaterials alternative to polymers, with more favorable therapeutic performance including antibacterial activity and fewer side effects.^8^

Inorganic oxide glasses such as silicate-based glasses, traditionally used for bone regeneration,^9^ have been recently proposed as materials for soft tissue regeneration and wound healing.^10^ In particular, silicate-based glasses in the system SiO_2_–P_2_O_5_–CaO–Na_2_O (45S5 Bioglass) have been shown to stimulate the secretion of growth factors and promote angiogenesis in vitro, with great potential for wound healing.^11^ When doped with metallic ions, *e.g.*, Ag^+^, silicate-based glasses also demonstrate antibacterial properties.^12^ Silicate-based glass fibers combined with polymers, *e.g.* poly(ε-caprolactone), have shown enhanced cell adhesion and proliferation properties.^13^ Borate-based glasses have also been proposed as alternatives to silicates due to their improved ion release properties and more rapid wound healing; however, they cannot be easily made at room temperature and toxicity is often a concern due to the release of borate ions.^14^

Phosphate-based glasses (PG) in the system P_2_O_5_–CaO–Na_2_O, traditionally used for hard tissue regeneration, have recently gained great interest because of their potential application in the regeneration of soft tissues.^15−19^

Unlike silicate- and borate-based glasses, PG are completely soluble in body fluids, and in contrast to polymers, PG leave no crystalline residues that could cause inflammation. Since they are bioresorbable, they are able to react and dissolve in physiological environments, thus being totally replaced with regenerated tissue. 45S5 silicate-based bioglass made via SG has been shown to have some skin healing properties in rats only after 10 days, with no effect of wound closure after 48 h.^20^ Additionally, borate SG cotton-like glass fibers have shown positive effects in wound healing;^21^ however, a recent cell viability study indicated that an increase in borate and calcium ions from borate SG fibers led to a decrease in cell viability.^22^ Solubility and ion release of PG can be controlled and tailored according to a specific application by altering the glass composition.^23,24^ PG are therefore ideal materials for controlled delivery of therapeutic species,^25^ in particular of therapeutic metallic ions (TMI) such as Cu^2+^,^26^ Ag^+^,^27^ Ca^2+^, and Zn^2+^^28^ or biologically active molecules (*e.g.* growth factors). PG also have many hydroxyl surface groups available for functionalization, which widens their range of applications. Note, TMI appear important for the regulation of wound repair. They are dynamically altered in wound tissue *in vivo* during normal healing, and deficient in delayed healing diabetic wounds. Thus, release of TMI from PG has considerable potential in terms of wound healing. Wound healing is associated with 4 main stages: hemostasis (up to 24 h), inflammation (∼ 4 days), proliferation (up to ∼ 21 days), and remodeling (months to years or longer; in patients affected by chronic wounds where wounds could never heal).^29^ Timelines are only indicative because they can vary with the type of wound and stages can overlap. For acute wounds and burns, times of each stage can be shorter: hemostasis (up to 6 h), inflammation (up to 24 h), proliferation (∼ 3–7 days), and remodeling (∼ 7–21 days).^30^ Various TMI have been found to be effective in promoting specific stages of wound healing (hemostasis, inflammation, proliferation, and remodeling). For wound healing applications, PGF offer many advantages over bulk PG. Their high surface area to volume ratio and open porosity promote gaseous exchange, removal of exudate, and cell migration.^31,32^ Moreover, an interconnected fibrous network similar to the structure of natural extracellular matrix supports cell attachment and proliferation.^33^ PGF are usually prepared via the traditional melt-spinning technique (MS) where oxide powders are melted at high temperatures (>1000 °C) followed by drawing fibers in a rotating drum.^15^ However, MS often leads to non-homogeneous glasses, incompatible with temperature sensitive molecules; in addition, manufacturing of porous PGF is not straightforward. Moreover, the viscosity of the glass, affected by the temperature, needs to be carefully controlled to avoid crystallization and reduction of metallic ions.^34,35^ The loss of volatile phosphorus during glass melting also leads to a difficult control of glass composition.^36^

Despite these limitations, PGF prepared via MS have been investigated for the engineering of fibrous soft tissues (muscles, ligaments, nerve conduits) where they guide cell growth and migration, fundamental steps in wound healing.^31^ The literature on the use of PGF for wound treatment is limited to a few studies on melt-derived fibers doped with Cu^2+^, Ga^3+^, and Ce^4+^.^15,37^

In this work, PGF have been produced by electrospinning (ES) of polyphosphate gels prepared via coacervation. The coacervation method is an environmentally friendly process that occurs at room temperature, in aqueous solution; it involves a dropwise addition of a divalent cation solution (*e.g.* Ca^2+^) to a solution of sodium polyphosphate (Na(PO_3_)*_n_*) that leads to the formation of a gel-like phase (coacervate).^38^ The coacervate gel is then used as an injectable precursor to produce PGF via ES. ES is an excellent green and cost-effective technique for making PGF alternative to the traditional MS.^8^ The ES process is particularly suitable to produce fibers as a dressing or matrix material for wound healing applications.^8,31,33,39^ ES produces flexible PGF easily conformable to complex wound shapes and sizes, and temperature sensitive therapeutic molecules can be incorporated. Unlike ES of most polymers (*e.g.* chitosan, cellulose, or gelatin), ES of phosphate-based coacervate gels does not require toxic or acidic solvents.^40^ Recently, coacervate PGF in the system P_2_O_5_–CaO–Na_2_O containing 1, 3, and 5 mol % CuO, demonstrated antibacterial activity against *S. aureus* and *E. coli*.^41^ PGF-Cu were investigated for their potential in bone regeneration and biocompatibility tested toward MG63 osteosarcoma cells. More recently, phosphate coacervate bulk gels in the system P_2_O_5_–CaO–Na_2_O with the incorporation of 0.1, 0.3, and 0.75 mol % Ag_2_O were tested for wound healing applications, showing an increase in viability of human skin cells (keratinocytes, HaCaTs) and antibacterial activity against non-antimicrobial resistant and antimicrobial resistant strains of Gram-positive (*S. aureus*, *Enterococcus faecalis*) and Gram-negative bacteria (*E. coli* and *Pseudomonas aeruginosa*).^42^

In this paper, we have investigated PGF in the system P_2_O_5_–CaO–Na_2_O containing 1, 2, 4, 6, and 10 mol % of Ag^+^, Zn^2+^, and Fe^3+^. The ternary system P_2_O_5_–CaO–Na_2_O has also been investigated for comparison. In particular, the therapeutic effect of these TMI in wound healing promotion has been investigated. The proposed PGF are designed to be multifunctional biomaterials alternative to current materials, with more favorable therapeutic performance and fewer side effects.

In particular, release studies are of interest to investigate the release of specific species that could contribute to different stages of wound healing.

Even though Ag^+^ has been known for a long time for its antibacterial properties, its ability to promote cell regeneration in wounds while simultaneously delivering an antibacterial effect has not been widely explored. There are reports of silicate-based glasses added with Ag^+^ for cariostatic applications,^43^ for antibacterial activity against *P. aeruginosa* and *S. aureus* in an *ex vivo* skin wound biofilm model,^12^ and for antibacterial effects against *S. aureus* in coacervate-based PG.^40^ Zinc phosphate coatings and mineralized guided bone regeneration membranes have been shown to reduce the adhesion of *E. coli* and inhibit colonization of *Aggregatibacter actinomycetemcomitans*, respectively.^44,45^ Zn^2+^ has also been shown to promote angiogenesis^46^ and cell proliferation,^47^ which contribute to wound healing. The therapeutic role and antibacterial activity of Fe^3+^ have also been reported. Iron oxide nanoparticles were reported to be antibacterial against *P. aeruginosa*(48) and *S. aureus*, and have antifungal properties.^49^ Iron oxide-containing MQ PG scaffolds have been shown to promote attachment and proliferation of myoblast cells for tissue engineering of skeletal muscle.^50^ Fe^3+^-doped MQ PG also show excellent biocompatibility, are non-toxic to human osteosarcoma cells,^51^ and have been demonstrated to reduce the solubility of the glass, hence slowing down the rate of ion release.^35^ Fe^3+^ has been reported to be beneficial in the wound healing process^52^ with an important role in promoting differentiation (*in vitro*) and wound epithelialization and extracellular matrix deposition in human skin (*ex vivo*).^53^ The role of endogenous iron in the body is well reported where the amount of iron in healing skin wounds in mice increases as healing progresses. By contrast, there is significantly lower iron content found in delayed healing diabetic wounds.^54^

In this study, keratinocyte scratch assays and *ex vivo* human skin biopsies were used to assess the wound healing promotion of PGF dissolution products in deionized water while varying the loading of each TMI (Ag^+^, Zn^2+^, and Fe^3+^) from 0 to 10 mol %. Antimicrobial efficacy was determined against planktonic strains of *S. aureus* and *E. coli* while human keratinocytes (HaCaTs) were used to assess biocompatibility. Ion release studies have been performed over time to establish if the TMI release is compatible with a suitable therapeutic range of concentrations. Our results have identified the most promising PGF systems (type and TMI loading) in terms of wound closure, antibacterial activity, or both.

## 2 Materials and Methods

### 2.1 Synthesis of Phosphate Coacervate Gels

20 mL of a 2 M aqueous solution of calcium nitrate tetrahydrate (Ca(NO_3_)_2_·4H_2_O, Acros, 99.0%) were slowly added to an equal volume of 4 M aqueous solution of sodium polyphosphate (Na(PO_3_)*_n_*, Merck, 99.0%), using a syringe pump (20 mL h^–1^) while stirring. Upon slow addition of the divalent cation Ca^2+^, a phase separation occurred forming an upper aqueous layer and a lower coacervate gel-like layer.^38,55^ To prepare samples with 1, 2, 4, 6, and 10 mol % of Ag^+^, Zn^2+^, and Fe^3+^, 0.6, 1.2, 3.6, and 6.0 mL of 2 M aqueous solutions of silver nitrate (AgNO_3_, Alfa Aesar, 99.9%), zinc nitrate hexahydrate (Zn(NO_3_)_2_·6H_2_O, Sigma-Aldrich, 99.0%), and iron nitrate nonahydrate (Fe(NO_3_)_3_·9H_2_O, Sigma-Aldrich, ≥98%) were added dropwise to the mixture, respectively, and stirred for a further hour to ensure homogenization. The mixtures were left to settle overnight at room temperature and then the supernatant was discarded, and the bottom coacervate gel layers were transferred into plastic syringes for ES.

### 2.2 Electrospinning of Phosphate Coacervate Gels

ES was performed at room temperature using a Spraybase system (Kildare, Ireland). A stainless-steel nozzle (gauge 18) was used, with a distance between the nozzle and the metallic plate collector of 15 cm, a flow rate of 2.0 mL h^–1^, and a voltage of 16 kV applied between the nozzle and the collector.

### 2.3 Characterization

Scanning electron microscopy (SEM) was performed using an Apreo 2 SEM (Thermo Fisher Scientific, Waltham), and the elemental composition was determined using energy-dispersive X-ray spectroscopy (EDX), attached to the SEM.

Raman spectroscopy was performed using a DXR3 Raman microscope (Thermo Fisher Scientific, Waltham). A wavelength of 532 nm and a power of 10 mW was used for all measurements over 24 scans and an exposure time of 3 s in the range 250–1350 cm^–1^.

X-ray diffraction (XRD) was performed using a PANalytical X’Pert spectrometer (Royston, UK) in a plate geometry using Ni-filtered Cu Kα X-ray radiation, with a wavelength of 1.5418 Å. Data was collected using a PIXcel-1D detector with a step size of 0.0525° and a time per step of 1.8 s over an angular range of 2θ = 20–90°.

### 2.4 Dissolution Studies

Ion release was assessed upon dissolution of the PGF in deionized (DI) water. 10 mg of each PGF were immersed in 10 mL of DI water and left in the solution for 3, 24, 48, and 72 h. Dissolution assays were performed in triplicate (*n* = 3). At each time point, the resulting suspensions were centrifuged at 4800 rpm for 5 min to separate the undissolved PGF from the solutions. Dissolution products were then filtered with 0.45 μm unit filters (Millipore filter unit, Millex-GP) and diluted 1:50 with 2% v/v nitric acid (HNO_3_ for trace metal analysis, Fisher Chemical), prior to analysis using microwave plasma atomic emission spectroscopy (MP-AES, Agilent 4210). Calibration standards for P, Ca, Na, Ag, Zn, and Fe were prepared from commercial stock solutions in 2% v/v HNO_3_, from which a linear calibration of 0.1, 0.5, 1, 2.5, 5, 10, 25, and 50 ppm concentrations was performed. The following wavelengths were used for each element; Na 588.95 nm, Ca 422.67 nm, P 213.618 nm, Ag 328.068 nm, Zn 481.053 nm, and Fe 371.993 nm. The signals were blank corrected using the 2% v/v HNO_3_ signal and normalized by an internal beryllium standard (5 ppm, 234.86 and 313.04 nm).

### 2.5 Antibacterial Studies

The antimicrobial effect of PGF’s dissolution products in DI water was assessed against a Gram-positive strain, *S. aureus* NCTC 8325, and a Gram-negative strain, *E. coli* K12. Both strains were cultured in Tryptic Soy Broth (TSB, Oxoid) and orbitally shaken at 250 rpm at 37 °C for 16–24 h. 1 μL of the resulting overnight bacterial culture was then incubated with 5 μL of dissolution product and 95 μL of TSB in a 96-well plate which was placed into a CLARIOStar plate reader (BMG Labtech) and incubated at 37 °C for 24 h. Absorbance was measured at 600 nm. Tests were performed in three biological replicates (*n* = 3) for each sample, and the undoped PGF was used as a negative control.

### 2.6 Cytocompatibility

The cytocompatibility of all PGF was assessed using the CellTiter 96 AQueous One Solution Cell Proliferation Assay (MTS). An immortalized cell line of human keratinocytes (HaCaTs, in vitro spontaneously transformed keratinocytes from histologically normal skin, AddexBio, Catalog Number T0020001, San Diego) was used for in vitro testing.

HaCaTs were cultured in high-glucose Dulbecco’s modified Eagle’s medium (DMEM) containing 0.4 mM calcium chloride (CaCl_2_ Gibco, Thermo Fisher Scientific, UK) with 10% v/v fetal bovine serum (Gibco) and 100 μg mL^–1^ penicillin-streptomycin solution (Gibco) in a humidified incubator at 5% CO_2_ and 37 °C. HaCaTs were seeded into 96-well plates at a density of 1 × 10^4^ cells per well and incubated with medium before adding 1% (v/v) of PGF dissolution product to the media. For this study, the PGF dissolution products used were obtained following 24 h contact between PGF and DI water. After incubation, the media containing the dissolution products was aspirated with fresh media and CellTiter MTS reagent added to each well as per manufacturer’s instructions. In this assay, the MTS tetrazolium compound is reduced in metabolically active cells to a soluble colored product (formazan). Therefore, the color change observed is directly proportional to the number of metabolically active cells in culture. Assay plates were incubated for 3 h at 37 °C and 5% CO_2_ and color change was measured on a spectrophotometer (Multiskan FC microplate reader; Thermo Fisher Scientific, United States) at a wavelength of 492 nm. Percentage change in cell viability was deduced from an untreated control.

### 2.7 Scratch Assays

Confluent monolayers of HaCaTs were scratched with a sterile 1 mL filter tip and treated with 1% (v/v) of each PGF dissolution product in growth media (as above) and incubated for 24 h. Scratches, stained with 1% crystal violet, were imaged (Nikon E400 microscope) and analyzed using ImageJ (National Institutes of Health, United States). The open scratch length was measured at five fixed points for each image with seven images taken for each well. Percentage closure of scratch wounds was determined at 24 h post wounding using a 0 h control.

### 2.8 Human *Ex Vivo* Wound Model and Whole-Mount Staining

The human *ex vivo* wound model and whole-mount staining approach used were based on the procedure described by Wilkinson et al.^56^ Human skin was obtained from patients undergoing reconstructive surgery at Castle Hill Hospital (“healthy” skin) and limb amputations at Hull Royal Infirmary (“chronic wound” skin). Skin was collected under fully informed, written patient consent, institutional guidelines, and ethical approval (LRECs: 17/SC/0220 and 19/NE/0150). Briefly, 2 mm partial thickness wounds were created in the center of 6 mm skin explants and cultured on a stack of absorbent pads and a 0.45 μm nylon filter at the air:membrane interface in 60 mm Petri dishes. Growth media consisting of standard DMEM containing 10% FBS and 1% penicillin/streptomycin solution (Gibco) was added to each Petri dish. Growth medium alone with no dissolution products was used as control. 1% (v/v) solutions of each PGF filtered 24 h dissolution product was added to growth media, with treated media applied topically to wounds. Wound explants were cultured at 35 °C and 5% CO_2_ for 2 days before collecting in neutral buffered formalin. Wound explants were stained with antimouse keratin 14 antibody (clone: LL002; Abcam) to measure wound closure (re-epithelialization) and counterstained with 1 μg/mL 4′,6-diamidino-2-phenylindole (Thermo Fisher Scientific). Keratin 14 was detected using Alexa Fluor 488-conjugated goat antimouse secondary antibody (Thermo Fisher Scientific). Wounds were imaged on a confocal laser scanning microscope (LSM 710, Carl Zeiss) using a 2.5× objective, 405 nm diode, and 488 nm argon lasers. Percentage closure was deduced as per our published method.^50^ A one-way ANOVA with Dunnett’s and Tukey post hoc was performed on *ex vivo* data, with significance determined where *p* < 0.05.

### 2.9 Statistical Analysis

All data are presented as mean ± standard deviation (SD), with at least three samples of each test. One-way analysis of variance (ANOVA) followed by Dunnett’s multiple comparison post hoc test was used for statistical analysis using GraphPad Prism software. Statistical differences were indicated with *p < 0.05 and **p < 0.01.

## 3 Results and Discussion

### 3.1 Synthesis of PGF

**Figure 1:**
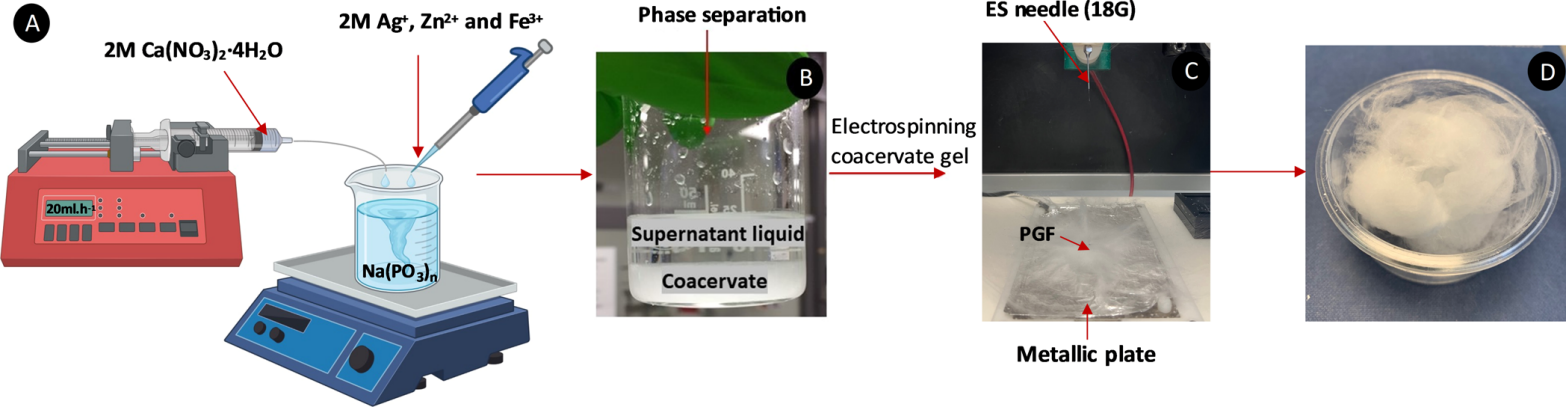
Schematic of PGF synthesis: (A) addition of Ca^2+^ to a sodium polyphosphate solution followed by addition of Ag^+^, Zn^2+^, or Fe^3+^; (B) phase separation with formation of the coacervate gel (bottom layer); (C) ES of coacervate gels and deposition of PGF on metallic plate; (D) “cotton-like” PGF (ternary system).

A schematic of the process used to synthesize PGF via ES of phosphate-based coacervate gels is shown in Figure 1. For the synthesis of the ternary PGF, an aqueous solution of Ca^2+^ was slowly added (20 mL h^–1^) to an aqueous solution of sodium polyphosphate (Na(PO_3_)_n_) and left stirring over 1 h (Figure 1A). For the doped samples, a similar process was used with Ag^+^, Zn^2+^, or Fe^3+^ ions added to the Na(PO_3_)*_n_* and Ca^2+^ mixture (after this has been stirred for 1 h) and stirred for a further hour, and allowed to settle overnight. As a result, a phase separation occurred with the coacervate gel layer at the bottom and an aqueous layer at the top (Figure 1B). After 24 h, the coacervate gel was loaded into a syringe and injected into an 18G nozzle to be electrospun on a metallic plate collector (Figure 1C), producing cotton-like PGF (Figure 1D). All PGF are amorphous, as demonstrated by the absence of Bragg peaks in the XRD patterns reported in Figure S1. The broad halo observed at around 2θ = 28° is due to the amorphous phosphate network. Therefore, the introduction of up to 10 mol % of Ag^+^ and Zn^2+^ and 4 mol % Fe^3+^ does not induce crystallization.

### 3.2 Assessment of PGF Composition

The elemental composition of PGF was assessed using SEM equipped with an EDX detector (atomic % of each element are reported in Table S1). Four sets of PGF were prepared: a ternary P_2_O_5_–CaO–Na_2_O system and three quaternary sets obtained by adding various amounts of Ag^+^, Zn^2+^, and Fe^3+^ to the ternary system. A final target loading of 1, 2, 4, 6, and 10 mol % of Ag^+^, Fe^3+^, and Zn^2+^ was attempted. However, coacervate gels containing 6 and 10 mol % of Fe^3+^ could not be electrospun into fibers due to their high viscosity. Compositions of PGF expressed in terms of mol % of oxides are reported in Table 1. The P_2_O_5_ content (46.6–48.6 mol %) and CaO content (34.5–42.1 mol %) were chosen on the basis of previous results on MQ and SG bulk systems that showed good bioactivity for glasses containing P_2_O_5_ in the range 45–50 mol % and CaO in the range 35–40 mol %.^57,58^ TMI content was also chosen on the basis of the literature on PGF.^59^ Samples will be hereafter named PGF-M-X, where M = Ag^+^, Zn^2+^and Fe^3+^ and X the nominal loading in mol % of Ag^+^, Zn^2+^, and Fe^3+^. The P_2_O_5_–CaO–Na_2_O system will be named PGF-ternary.

**Table 1:** Elemental Analysis of PGF Expressed as Oxide mol %, Measured by EDX

	oxide composition (mol %)					
sample	P_2_O_5_	CaO	Na_2_O	Ag_2_O	ZnO	Fe_2_O_3_
PGF-ternary	47.8 ± 2.1	41.3 ± 1.9	10.9 ± 0.45			
PGF-Ag-1	47.6 ± 4.7	41.6 ± 5.1	10.2 ± 0.3	0.6 ± 0.1		
PGF-Ag-2	47.8 ± 2.4	39.5 ± 2.0	11.7 ± 0.4	1.0 ± 0.1		
PGF-Ag-4	48.0 ± 7.8	41.1 ± 7.8	9.0 ± 0.6	2.0 ± 0.4		
PGF-Ag-6	48.6 ± 3.5	38.9 ± 4.0	9.4 ± 1.2	3.1 ± 0.2		
PGF-Ag-10	48.5 ± 1.1	40.3 ± 0.7	7.5 ± 0.4	3.7 ± 0.1		
PGF-Zn-1	47.9 ± 1.9	40.2 ± 2.1	10.6 ± 0.4		1.3 ± 0.1	
PGF-Zn-2	47.3 ± 2.8	38.7 ± 2.6	11.9 ± 0.2		2.0 ± 0.2	
PGF-Zn-4	46.9 ± 2.0	36.7 ± 2.1	12.4 ± 0.5		4.0 ± 0.2	
PGF-Zn-6	46.8 ± 2.6	37.4 ± 3.0	10.3 ± 0.4		5.5 ± 0.3	
PGF-Zn-10	46.6 ± 2.0	34.5 ± 1.6	9.6 ± 1.4		9.3 ± 0.6	
PGF-Fe-1	47.8 ± 0.8	40.8 ± 2.1	10.6 ± 1.1			0.8 ± 0.1
PGF-Fe-2	48.0 ± 6.2	37.3 ± 7.7	13.5 ± 1.1			1.2 ± 0.1
PGF-Fe-4	48.0 ± 1.2	42.1 ± 1.9	8.2 ± 0.4			1.6 ± 0.1

EDX chemical mapping indicates that all elements are homogeneously distributed on the surface of PGF as shown in the representative chemical map of all elements for the PGF-Ag-10 sample reported in Figure S2.

### 3.3 Assessment of PGF Morphology

**Figure 2:**
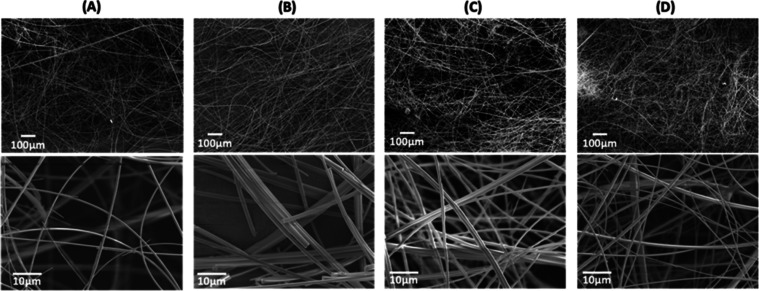
SEM representative images of (A) PGF-ternary, (B) PGF-Ag-2, (C) PGF-Zn-2, and (D) PGF-Fe-2 quaternary PGF at 100x (top) and 2000x (bottom) magnifications.

SEM images of PBF-ternary and PGF-Ag, PGF-Zn, and PGF-Fe are provided in Figure 2 (representative samples containing 2 mol % of TMI) and Figure S3 (all samples). The average diameter of the PGFs, measured using ImageJ (NIH, US) from 20 individual fibers, varied within each sample, ranging on average from about 1–5 μm as reported in Figure S4. Respresentative samples containing 2 mol % TMI demonstrated average diameters of PGF-Ag: 4.2 ± 1.4 μm, PGF-Fe: 5.2 ± 2.5 μm, PGF-Zn: 1.4 ± 0.9 mm, with PGF-Zn having the lowest average diameter of all PGF. Evaluation of PGF diameter is important as it has been reported that the degradation rate of PGF increases with decreasing fiber diameter for a given mass of fiber due to the increased surface area to volume ratio.^15^

### 3.4 Raman Spectroscopy

**Figure 3:**
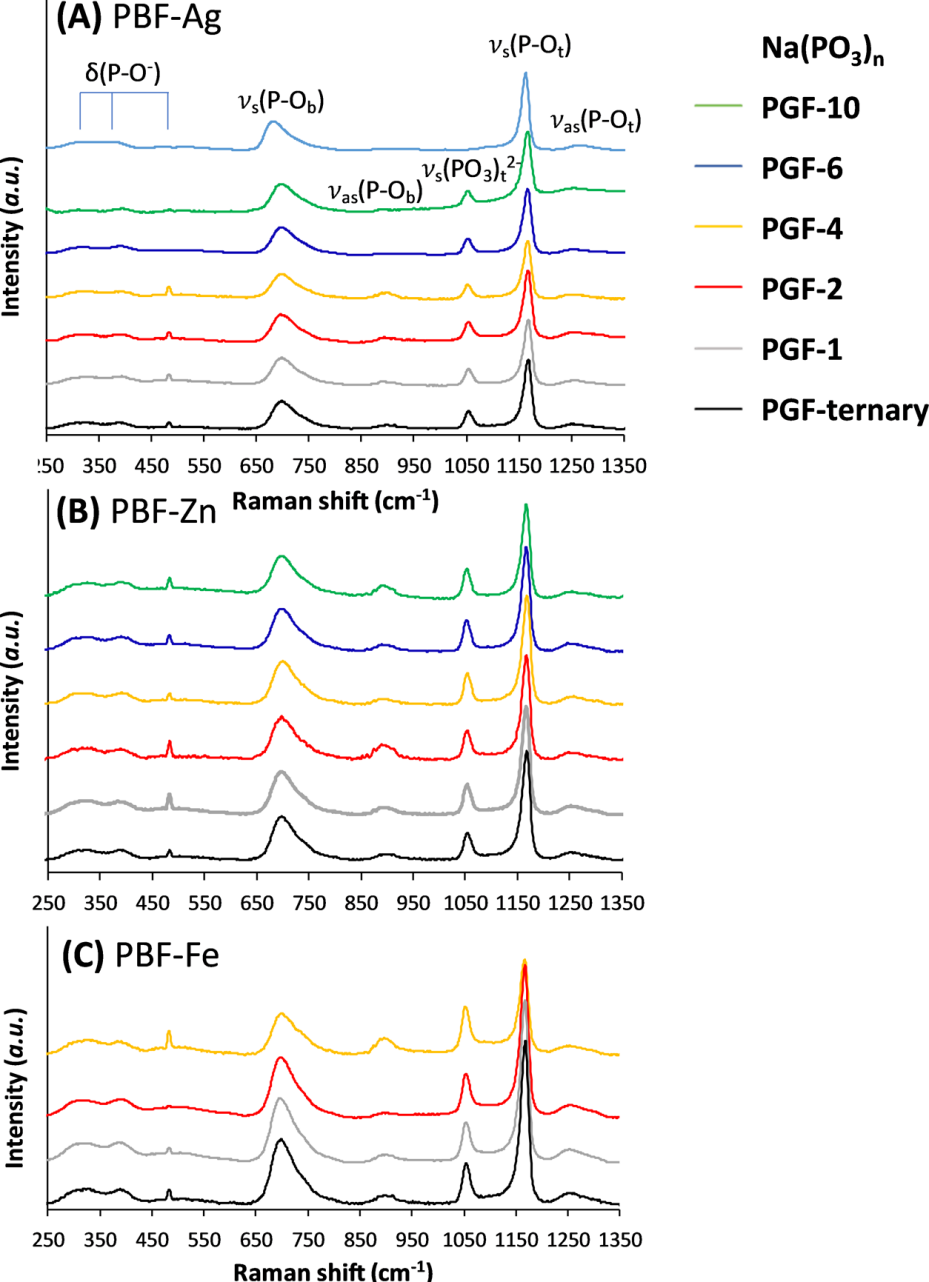
Raman spectra of (A) PGF-Ag, (B) PGF-Zn, and (C) PGF-Fe. Raman spectra of the ternary PGF and Na(PO_3_)*_n_* reference are also shown.

The structure of the PGF network was investigated using Raman spectroscopy. The PO_4_^3–^ tetrahedra units will be described using the Q^n^ notation, where n indicates the number of bridging oxygens. Raman spectra of PGF-Ag, PGF-Zn, and PGF-Fe and that of sodium polyphosphate Na(PO_3_)*_n_* are shown in Figure 3A–C, respectively. The spectra obtained are similar to those of bulk PG prepared via MQ and SG.^40,60−62^ No significant differences are observed by changing the Ag, Zn, or Fe loading within a series or between series. The bands at 325, 380, and 485 cm^–1^ are assigned to the bending vibrations (δ) of P–O^–^ groups (Q^1^), which are also observed in Na(PO_3_)*_n_*.^63^ The bands at 705 and 900 cm^–1^, present in all PGF, are assigned to the symmetrical and asymmetrical stretching modes (ν) of in-chain P–O–P units (P–O_b_, Q^2^ bridging units), respectively.^64^ The intense band at 1170 cm^–1^ and the broader band at 1250 cm^–1^ are due to symmetric and asymmetric stretching of the out-of-chain P–O_t_, respectively.^62,65^ A decrease in intensity for the two latter bands is observed for PGF-Fe as the Fe^3+^ loading increases (Figure 3C) indicating a decrease of out-of-chain P–O_t_, units. This suggests that Fe^3+^ has a strong chelating effect and allows more bridging oxygens and therefore a more interconnected system than the PGF-Ag and PGF-Zn systems. This is in agreement with a previous study on aluminum polyphosphate gels, where Al^3+^ was demonstrated to cross-link the polyphosphate chain.^66^ The band at 1050 cm^–1^ is attributed to the symmetric stretching mode of the (PO_3_)_t_^2–^ groups, related to the chain-terminating Q^1^ units. Interestingly, Na(PO_3_)*_n_* does not show the band at 1050 cm^–1^. Moreover, no strong peaks at around 950–1000 cm^–1^ are present indicating the absence of Q^0^ orthophosphate units which have no bridging oxygens.^67^

### 3.5 Ion Release Studies

**Figure 4:**
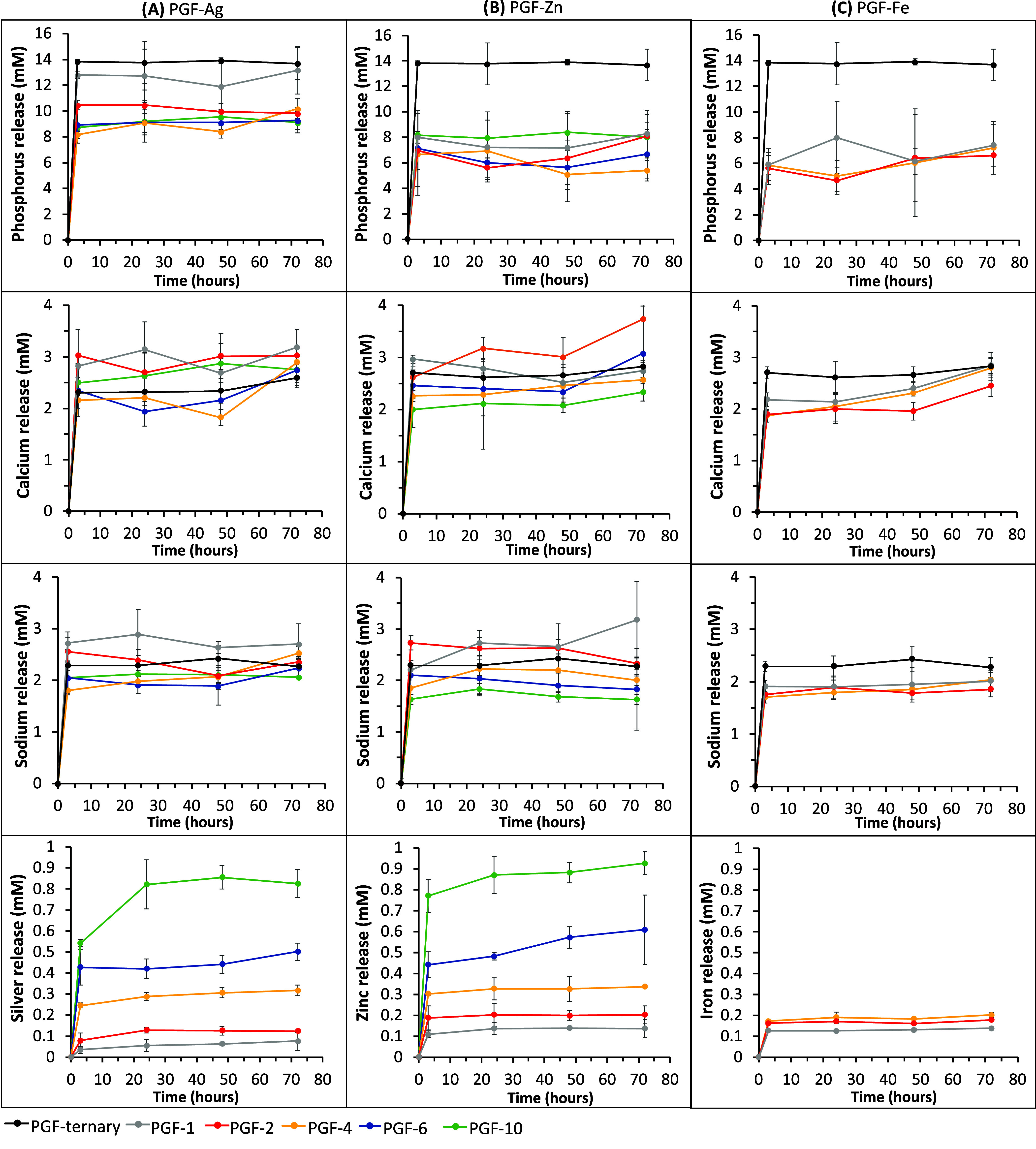
Release profiles of P (phosphate anions), Ca^2+^, Na^+^, Ag^+^, Zn^2+^, and Fe^3+^ after immersion in DI water up to 72 h from (A) Ag-PGF, (B) Zn-PGF, and (C) Fe-PGF. Error bars indicate mean ± SD (*n* = 3).

Given that PGF can find application as controlled delivery systems, dissolution studies are of paramount importance. The release profiles of P, Ca, Na, and TMI from PGF-Ag, PGF-Zn, and PGF-Fe in DI water over 72 h are shown in Figure 4A–4C, respectively.

The amount of phosphorus released is much higher for the undoped PGF compared to all other compositions. The overall amount of P released is higher for PGF-Ag than for PGF-Zn and PGF-Fe.

This result is in agreement with previous data showing a decrease in degradation (enhanced durability) in PGF containing Fe_2_O_3_ and ZnO.^15^

No significant trends were observed in the release of Ca^2+^ and Na^+^ with a change in TMI loading or between series. Similar concentrations of Ca^2+^, Na^+^ and phosphate anions were reported in previous studies on coacervate PGF containing Cu^2+^.^41,42^ However, another recent study investigating coacervate PG gels containing Ag^+^, demonstrated a greater release of phosphates, Ca^2+^, Na^+^, and Ag^+^ in comparison to the PGF presented in this work.^42^

The dissolution of Ag^+^, Zn^2+^, and Fe^3+^occurs mainly in the first hours of immersion in DI water, with an increase in TMI above 4 mol % slowing down the degradation closer to 24 h. The release of Ag^+^ and Zn^2+^ from the PGF rises significantly with increasing TMI loading. In particular, both PGF-Ag and PGF-Zn release similar concentrations of Ag^+^ and Zn^2+^ (in mM) for analogous samples within a series. At 24 h, the release of Ag^+^ and Zn^2+^ is ∼ 0.8 mM (10 mol % TMI), ∼ 0.4 mM (6 mol %, TMI), and ∼ 0.3 mM (4 mol % TMI).

The release of Fe^3+^ from PGF-Fe on the other hand, seems to be much less dependent on Fe loading, only slightly increasing from PGF-Fe-1 to PGF-Fe-4. For PGF-Fe-4, the release of Fe^3+^ is ∼ 0.2 mM at 24 h. At 24 h, the concentrations of Zn^2+^ released from PGF-Zn-2 and from PGF-Zn-1 are ∼ 0.2 mM and 0.1 mM, respectively, whereas those of Ag^+^ from PGF-Ag-2 and from PGF-Ag-1 are lower (∼ 0.1 and ∼ 0.05 mM). Fe^3+^ release is similar to Zn^2+^ at 1 and 2 mol %.

The effect of zinc and iron ions during full-thickness wound healing has been presented by Coger *et al*.^68^ A rise of zinc ion concentration from 8 h to 3 days in a healing wound (up to 40 ppm) induces the keratinocyte proliferation during the same time, as keratinocyte proliferation and differentiation are controlled by zinc. An increase in Fe ions concentration is also observed from day 1 to day 3 in healing wounds, with a maximum of 3 days at the peak of the proliferation phase. This indicates that our PGF releases a suitable Zn^2+^ concentration in a suitable time frame.

### 3.6 Antibacterial Studies

**Figure 5:**
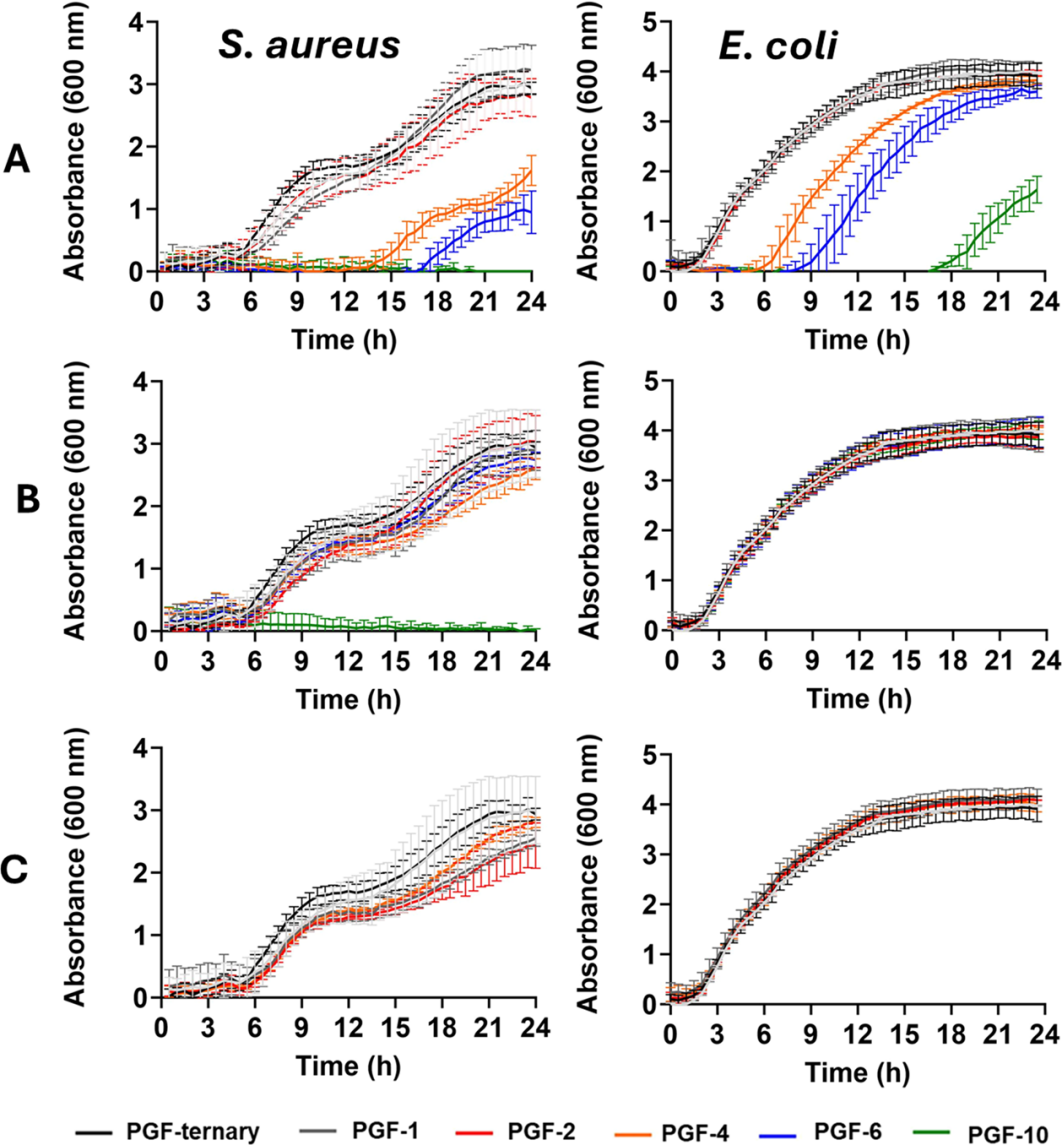
Antibacterial activity of (A) PGF-Ag, (B) PGF-Zn, and (C) PGF-Fe against *S. aureus* NCTC 8325 (left) and *E. coli* K12 (right) expressed as the mean ± SD (*n* = 3).

The antimicrobial activity of PGF-Ag, PGF-Zn, and PGF-Fe against *S. aureus* and *E. coli* was monitored using the optical density (OD) of the bacterial cultures. The OD reading is directly proportional to cell growth, therefore, ideal to record changes in bacterial growth when the samples were in contact with the dissolution products of PGF at 37 °C. The dissolution products were obtained after 24 h immersion of PGF in DI water (as per ion release studies). PGF-Ag showed a greater antibacterial efficacy (Figure 5A) in comparison with PGF-Zn (Figure 5B) and PGF-Fe (Figure 5C). Above 2 mol % of Ag^+^, the rate of bacterial growth of *S. aureus* and *E. coli* was significantly delayed (*p* < 0.05), and completely inhibited at a concentration of 10 mol %. Although similar concentrations of Ag^+^ and Zn^2+^ were released from the PGF products, Zn^2+^ only inhibited *S. aureus* growth at 10 mol %, with no inhibitory effect against *E. coli*. Silver is well known for its exceptional antibacterial properties, making it a widely used element for its bactericidal effects.^69,70^ The mechanism of bactericidal action of silver involves the release of Ag^+^, which binds to proteins and nucleic acids, inhibiting cell division and reproduction.^71^ The mechanism involves several pathways such as disruption of cell membranes, binding to cellular components, *e.g.* thiol groups, generation of reactive oxygen species, interference with DNA replication, and disruption of electron transport chain. These mechanisms collectively contribute to silver’s broad-spectrum antimicrobial activity, making it effective against a wide range of bacterial species.^72^ Compared to zinc, silver demonstrates superior bactericidal activity due to its higher ability to penetrate cell membranes and more effective attachment to bacterial surfaces.^73^ Mechanism of action of metallic ions on bacteria can be complex and some approaches to explain such mechanisms have been presented in the literature.^74,75^ PGF-Fe samples did not significantly affect the growth of both bacteria. This might be due to the low concentration of Fe^3+^ in solution as previously reported by Sun *et al.*(76) The bactericidal effect of Fe^3+^ against *E. coli* was only observed for concentrations greater than 1 mM, but no effect was observed at a concentration of 0.5 mM.

### 3.7 Cell Viability

**Figure 6:**
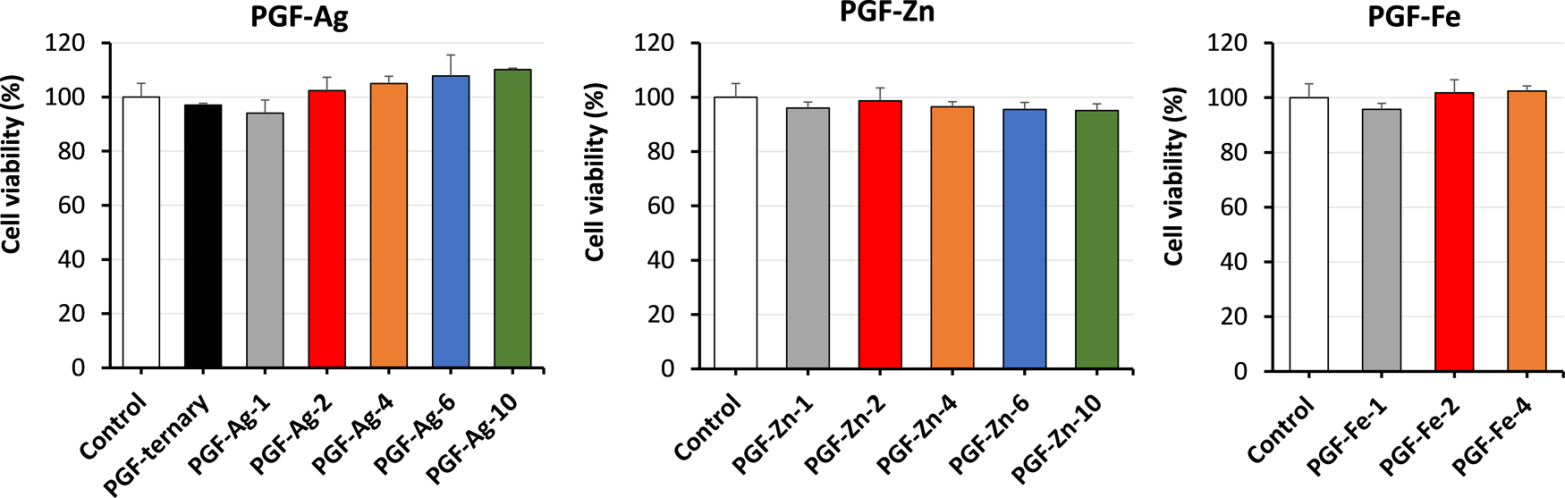
Average cell viability of HaCaTs (MTS assay) following 24 h treatment with PGF-Ag, PGF-Zn, and PGF-Fe dissolution products in DI water. Data show mean + SD (*n* = 3).

Given that the fibrous morphology of PGF-Ag, PGF-Zn, and PGF-Fe is well suited for the fabrication of wound dressing, their biocompatibility was assessed via an MTS assay using HaCaTs cells, an immortalized cell line of human keratinocytes (cells found in the epidermis) widely used in skin research.^77^ PGF cytotoxicity has been investigated through indirect tests, where the cells are in contact with the products resulting from PGF dissolution in DI water after 24 h, and not directly seeded on the PGF. The growth media without any dissolution product was used as a reference control. No significant difference in cell viability was observed between the control medium and the dissolution products at 24 h post-treatment. This result indicates that the dissolution products of all PGF after 24 h contact time between DI water and PGF powders are not cytotoxicity (Figure 6).

As discussed in the ion release section, at 24 h, the release of Ag^+^ and Zn^2+^ from PGF-Ag-10/PGF-Zn-10, PGF-Ag-6/PGF-Zn-6, and PGF-Ag-4/PGF-Zn-4 are ∼0.8, ∼0.4, and ∼0.3 mM, respectively. Zn^2+^ released from PGF-Zn-2 and PGF-Zn-1 are ∼0.2 and 0.1 mM, respectively, whereas Ag^+^ from PGF-Ag-2 and PGF-Ag-1 are ∼0.1 and 0.05 mM, respectively. At 24 h, PGF-Fe-4/PGF-Fe-2 release of Fe^3+^ is ∼0.2 mM and slightly lower for PGF-Fe-1. Cytocompatibility results show that all of the above TMI concentrations are safe to use in the presence of HaCaTs.

### 3.8 Scratch Migration Assay

**Figure 7:**
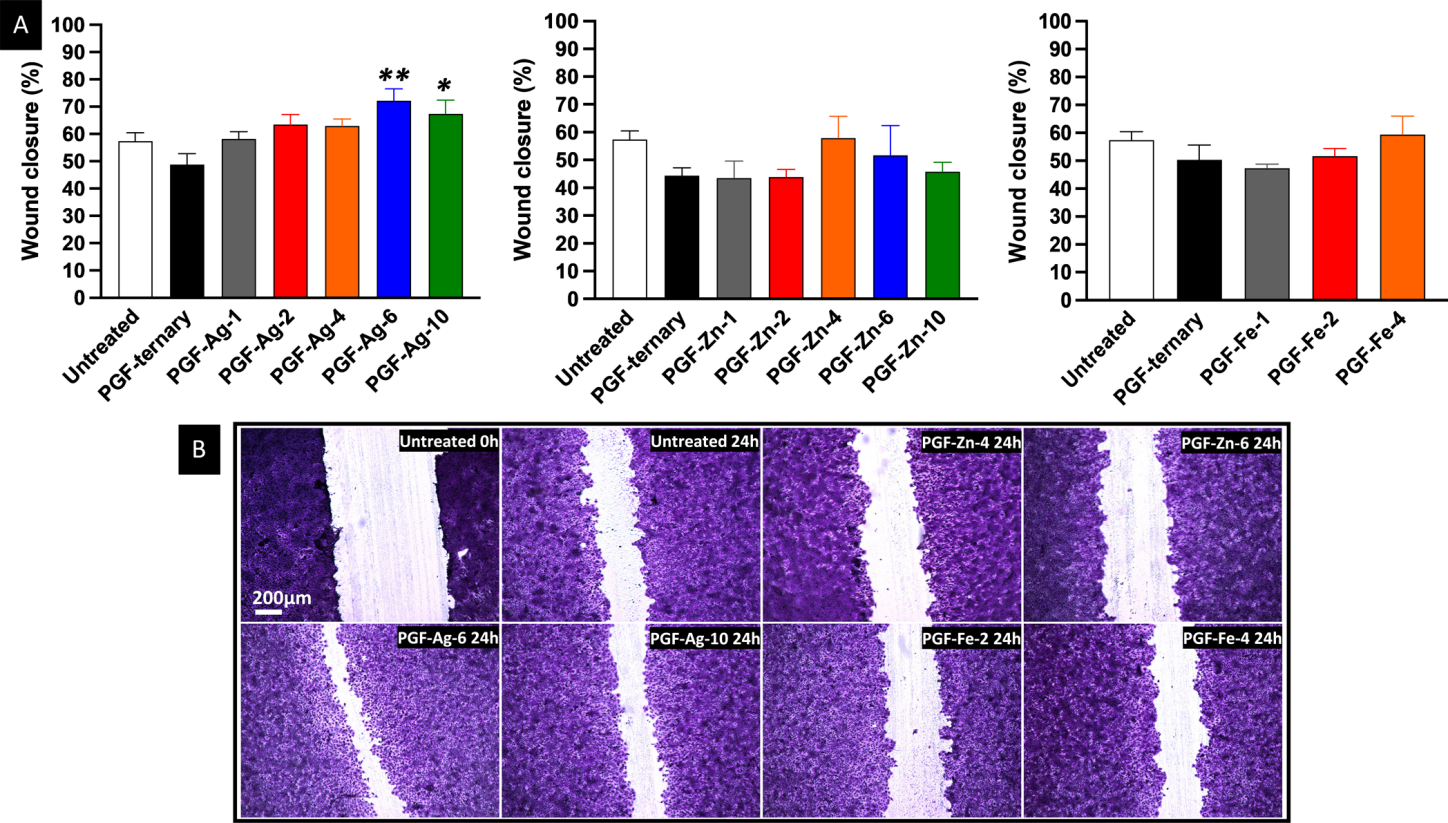
(A) Wound closure % of scratches performed on HaCaTs after 24 h treatment with dissolution products from PGF-Ag, PGF-Zn, and PGF-Fe. Mean + SD (*n* = 3). One-way ANOVA: *p < 0.05 and **p < 0.01. (B) Bright-field images of the scratch and HaCaTs in contact with the control (growth medium), untreated after 24 h, and after 24 h treatment with selected PGF-Ag, PGF-Zn, and PGF-Fe dissolution products.

To evaluate the wound healing performance of all PGF, a scratch assay was performed which enabled cell migration to be assessed on a confluent layer of HaCaTs. Histograms showing wound closure % for all samples and compositions are shown in Figure 7A. The wound partially heals naturally after 24 h with a wound closure of about 58%. However, a significant increase in wound closure after 24 h incubation with the PGF’s dissolution products with Ag^+^ greater than 1 mol % was observed. In particular, both PGF-Ag-6 and PGF-Ag-10 significantly increased % wound closure compared to the untreated 24 h control (67 and 72%, respectively). A similar effect has been reported with SG borate-based glasses containing Ag^+^, where keratinocyte scratch tests demonstrated significant wound closure enhancement with increasing silver content.^78^ However, no significant changes in wound closure rates were observed in HaCaTs treated with PGF-Zn or PGF-Fe. This could be explained considering that Ag^+^ has been previously reported to enhance the expression of keratinocyte growth factor 2, which stimulates the proliferation and migration of HaCaTs cells.^79^ It has also been reported that Ag^+^ increases the amount of intracellular reactive oxygen species, which may be the reason for increasing cell proliferation.^80^ For better visualization of wound closures, bright-field images of HaCaTs cells in contact with the control (growth medium) immediately after immersion (untreated 0 h), after 24 h (untreated 24 h), and in contact with the best-performing dissolution products of selected PGF after 24 h are shown in Figure 7B. It is evident that PGF-Ag-6 and PGF-Ag-10 are the best compositions for wound healing promotion.

### 3.9 Human Ex Vivo Skin Wounding Model

**Figure 8:**
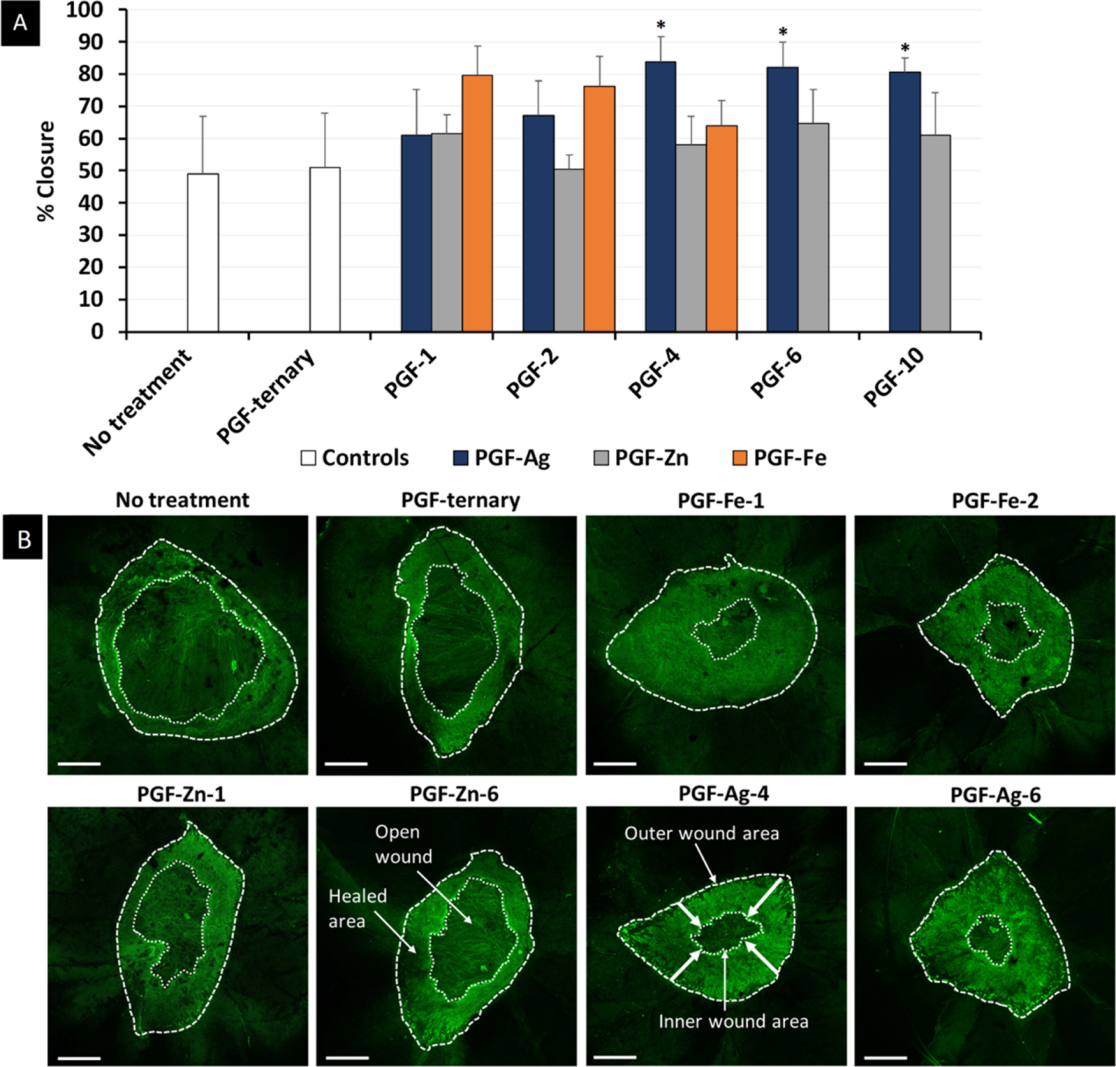
(A) Average % wound closure of healthy human skin after treatment with PGF dissolution products. Mean + SD (*n* = 3). One-way ANOVA followed by Dunnett’s post hoc test were used, where *p < 0.05. (B) Representative confocal images of healing over 48 h in healthy human skin after treatment with selected PGF-ternary, PGF-Ag, PGF-Zn, and PGF-Fe. “No treatment” was used as a growth media control on the skin (*n* = 3). Alexa Fluor 488 = Keratin 14. Bar = 500 μm.

The wound healing-promoting effects of PGF dissolution products were further confirmed using an established human *ex vivo* skin wounding and whole-mount staining approach.^50^ This model is more translationally applicable than scratch migration testing, enabling assessment of wound closure (re-epithelialization) in a native, living human skin environment. The average % wound closure of all PGF is shown in Figure 8A. The histograms confirm that the 24 h dissolution products of PGF-Ag are the most successful systems, in particular those from PGF-Ag-4 (84%), PGF-Ag-6 (82%), and PGF-Ag-10 (81% closure). PGF-Zn and PGF-Fe showed no statistically significant effect on wound closure. This is also confirmed by the confocal images of healing over 48 h reported in Figure 8B. A 48 h healing time was employed for the *ex vivo* study in comparison to 24 h for the in vitro scratch migration assay, since healing of a human skin biopsy would generally be slower than the closure of a scratch in a monolayer of HaCaTs, which typically close completely when incubating longer than 24 h. Both *in vitro* and *ex vivo* results in this paper similarly demonstrate the promotion of wound healing from PGF-Ag; however, our in vitro study demonstrated a positive effect only for Ag^+^ loading ≥6 mol %, whereas the *ex vivo* study demonstrated activity at lower loading, Ag^+^ loading ≥4 mol %. These differences can be attributed to variations in methodology, as in the former, the monolayer cells are wounded when cells are removed with a pipet tip and the latter involves excision of tissue via a partial thickness biopsy. The *ex vivo* method is more suitable to evaluate wound healing effects since it is more comparable to the real-world situation, exhibiting native tissue structure and resident primary skin cells.^81^

Despite its advantages over in vitro assays, the *ex vivo* model still lacks a circulation system, and therefore is largely limited to assessment of epidermal healing over a short time frame. By contrast, *in vivo* studies enable evaluation of other important aspects of healing, including immune cell recruitment, granulation tissue formation, and extracellular matrix remodeling.^82^ However, most *in vivo* wound healing studies are carried out in rodents, which generally exhibit limited translation to humans due to differences in skin structure and wound healing mechanisms.^83^ Therefore, future work should combine both *ex vivo* and *in vivo* approaches to enable detailed evaluation of wound healing effects (*in vivo*) and cross-validation of translational applicability (*ex vivo*).

**Figure 9:**
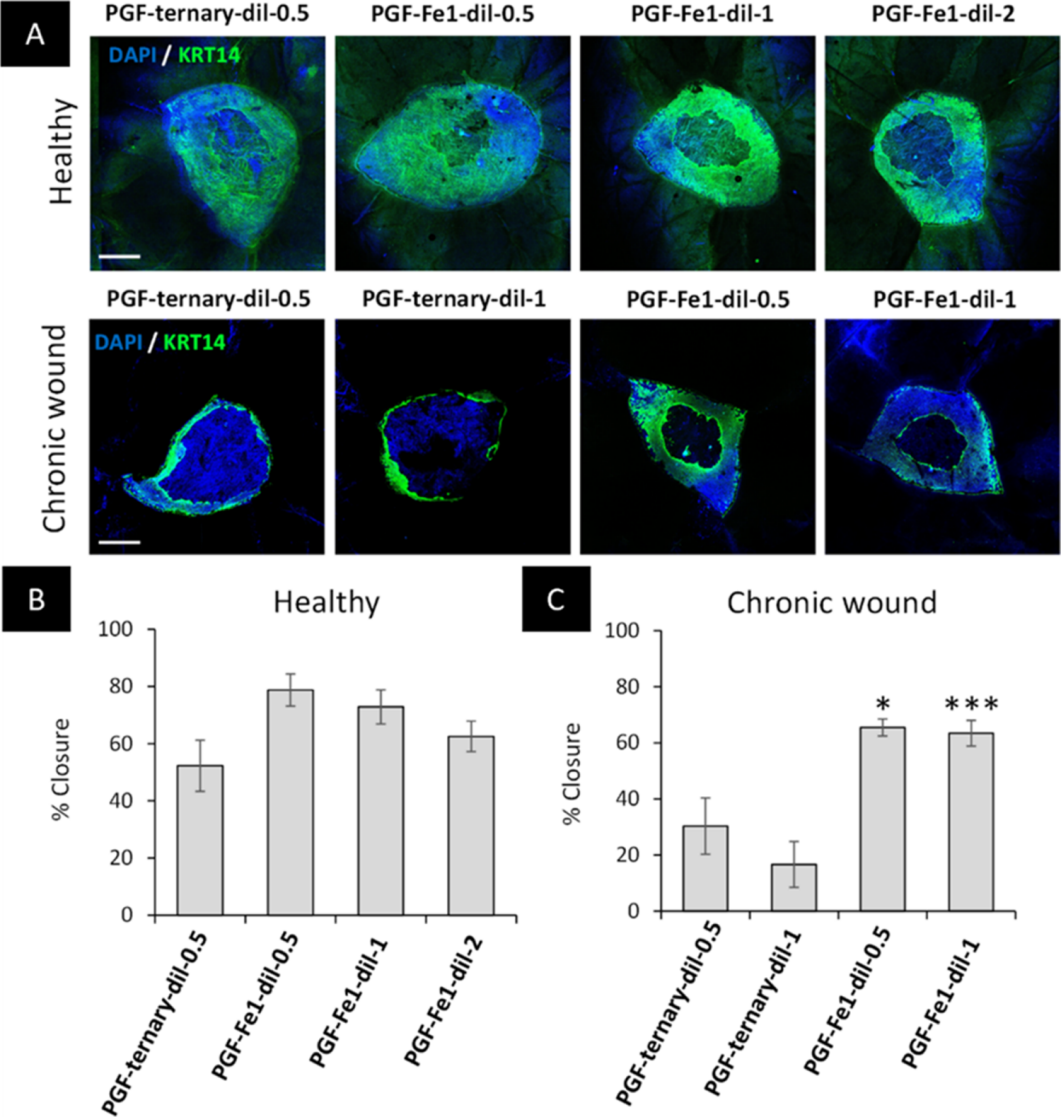
Fe-doped PGF significantly accelerates healing in chronic wounded skin. Representative confocal images (A) and percentage closure following 48 h PGF treatment in healthy (B) and chronic wounded (C) human skin. Scale bar = 500 μm. KRT14 = Alexa Fluor 488. One-way ANOVA with Tukey posthoc where *P < 0.05 and ***P < 0.001.

To further increase translational relevance, PGF-ternary and PGF-Fe-1 were then preliminary tested in skin from a patient with a chronic wound (Figure 9). Recently, the ex vivo wound model was used to demonstrate healthy skin wounds close significantly faster than *ex vivo* wounds created from chronic wound skin over 7 days.^50^ In our tests, we found comparable rates of healing, where control healthy skin healed at a faster rate than the chronic wound skin (52 vs 30%, respectively). Dilution factors presented (0.5, 1, and 2) refer to the dissolution product diluted into growth media. Although a non-significant increase in closure was observed in wounds from healthy skin (Figure 9A-top and B), the results clearly show that PGF-Fe significantly increases wound closure rates in chronic wound skin from 30 to 65% at 0.5% and 16–63% at 1% (Figure 9A-bottom and C), thus suggesting that PGF-Fe could be a promising strategy for treating non-healing wounds in a clinical setting.

## 4 Conclusions

PGF in the ternary P_2_O_5_–CaO–Na_2_O system undoped and added with Ag^+^, Zn^2+^ (up to 10 mol %), and Fe^3+^ (up to 4 mol %) were successfully synthesized via ES of coacervate gels. EDX analysis showed that all elements are homogeneously distributed on the surface of PGF. Raman spectra showed characteristic bands expected from phosphate chains formed predominately by Q^1^ and Q^2^ phosphate units. PGF dissolution studies showed that Ca^2+^, Na^+^, phosphate anions, and TMI are mainly released within the first 24 h in DI water, with the release of TMI increasing with the increase in TMI content. The addition of Zn^2+^ and Fe^3+^ to PGF slightly decreases the solubility of the phosphate chains. PGF-Ag showed antibacterial activity against both *S. aureus* and *E. coli* at Ag^+^ loading ≥ 4 mol %, while PGF-Zn was effective only against *S. aureus* at Zn^2+^ loading ≥10 mol %. PGF-Fe did not show any antibacterial activity. Cytocompatibility studies using human keratinocytes demonstrated that all PGF dissolution products are non-toxic. PGF containing ≥4 mol % of Ag^+^ demonstrated significant enhancement of wound closure in human *ex vivo* experiments (84%), while *in vitro* scratch migration study showed effective HaCaTs migration/proliferation for ≥6 mol % Ag^+^, reaching 72% increase in wound closure for the system containing 10 mol % of Ag^+^. Finally, PGF-Fe-1 was able to rapidly accelerate healing in chronic wound patient skin (>30%), suggesting that PGF containing Fe are promising materials for wound healing applications.

## Data Availability

Data will be made available upon request.

## Supporting Information Available

XRD patterns of PGF containing Ag^+^, Zn^2+^, and Fe^3+^; XRD patterns of the ternary PGF for comparison; elemental analysis of PGF expressed as atomic % of each element, measured by EDX; representative EDX mapping showing the elemental distribution of P, Ca, Na, O and Ag on the surface of PGF-Ag-10; SEM images of all PGF-Ag, PGF-Zn, PGF-Fe, and PGF-ternary; mean fiber diameter of PGF-ternary, PGF-Ag, PGF-Zn, and PGF-Fe, containing 1, 2, 4, 6, and 10 mol % of TMI (PDF)
