# Supplementary material for: Wound Healing Promotion via Release of Therapeutic Metallic Ions from Phosphate Glass Fibers: An In Vitro and Ex Vivo Study
Source: ACS Appl Mater Interfaces. 2024 Jul 16;16(29):37669–82. doi: 10.1021/acsami.4c07035 (PMC11284751; doi:10.1021/acsami.4c07035)
Supplement: Supplementary file 1 — am4c07035_si_001.pdf [file am4c07035_si_001.pdf]

Supporting Information

Wound healing promotion via release of therapeutic metallic ions from phosphate glass fibres: an in vitro and ex vivo study

Agron Hoxha [a], *Athanasios Nikolaou* [a, b], *Holly N. Wilkinson* [c,d], *Matthew J. Hardman* [c,d], *Jorge Gutierrez-Merino* [b], *Monica-Felipe Sotelo* [a], *Daniela Carta* [a,*]

[a] *School of Chemistry and Chemical Engineering, University of Surrey, Guildford, GU2 7XH, United Kingdom*

[b] *School of Biosciences and Medicine, University of Surrey, Guildford GU2 7XH, United Kingdom*

[c] *Centre for Biomedicine, Hull York Medical School, University of Hull, Hull HU6 7RX, United Kingdom*

[d] *Skin Research Centre, Hull York Medical School, University of York, York YO10 5DD, United Kingdom*

- E-mail: d.carta@surrey.ac.uk

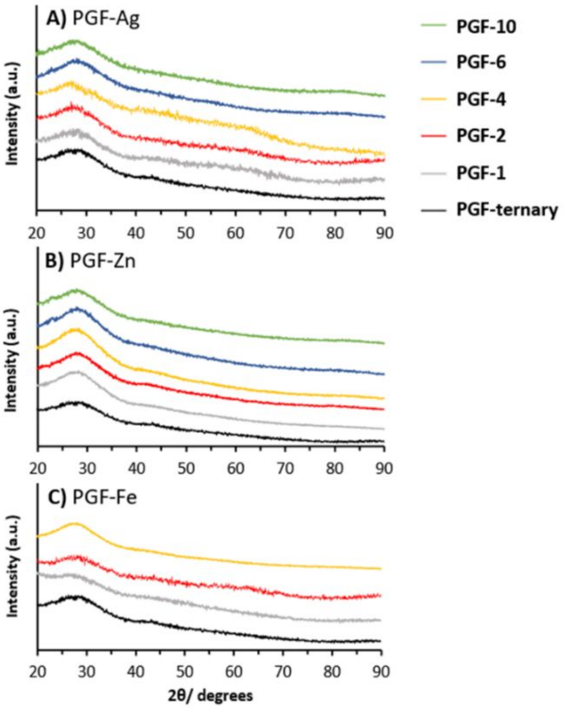

Figure S1. XRD patterns of PGF containing (A) Ag^{+}, (B) Zn^{2+}, and (C) Fe^{3+}. XRD patterns of the ternary PGF is also shown as comparison (black line).

Table S1. Elemental analysis of PGF expressed as atomic % of each element, measured by EDX.

Sample	Atomic %						
	O	Na	P	Ca	Ag	Zn	Fe
**PGF-ternary**	65.2	4.8	21.0	9.1	-	-	-
**PGF-Ag-1**	64.6	4.6	21.3	9.3	0.3	-	-
**PGF-Ag-2**	70.3	4.3	17.7	7.3	0.4	-	-
**PGF-Ag-4**	66.7	3.8	20.1	8.6	0.8	-	-
**PGF-Ag-6**	65.7	4.0	20.7	8.3	1.3	-	-
**PGF-Ag-10**	62.5	3.6	22.8	9.5	1.7	-	-
**PGF-Zn-1**	64.0	4.8	21.7	9.1	-	0.3	-
**PGF-Zn-2**	63.1	5.5	21.9	9.0	-	0.5	-
**PGF-Zn-4**	62.5	5.8	22.1	8.6	-	1.0	-
**PGF-Zn-6**	57.9	5.5	25.1	10.0	-	1.5	-
**PGF-Zn-10**	63.9	4.5	21.6	8.0	-	2.2	-
**PGF-Fe-1**	60.2	5.3	23.9	10.2	-	-	0.4
**PGF-Fe-2**	69.1	5.2	18.3	7.1	-	-	0.5
**PGF-Fe-4**	61.8	4.0	23.3	10.2	-	-	0.8

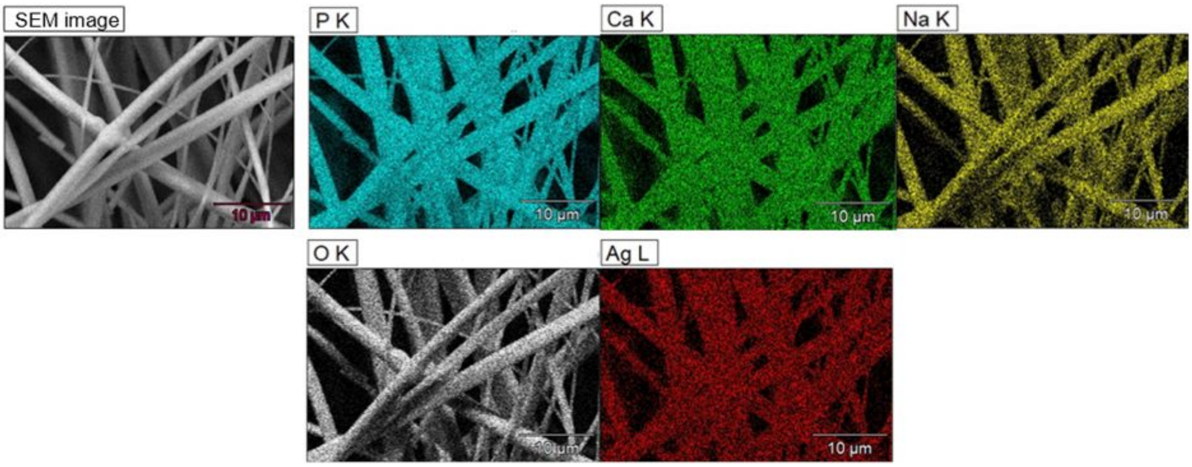

Figure S2. Representative EDX mapping showing the elemental distribution of P, Ca, Na, O and Ag on the surface of PGF-Ag-10.

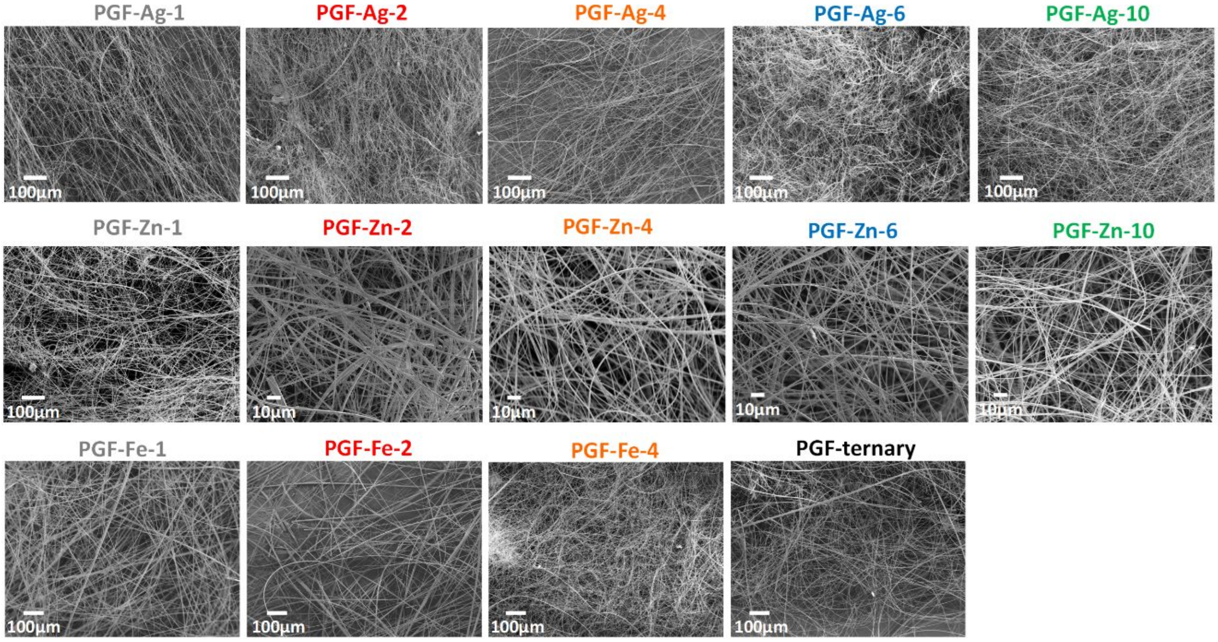

Fig. S3. SEM images of all PGF-Ag, PGF-Zn, PGF-Fe and PGF-ternary.

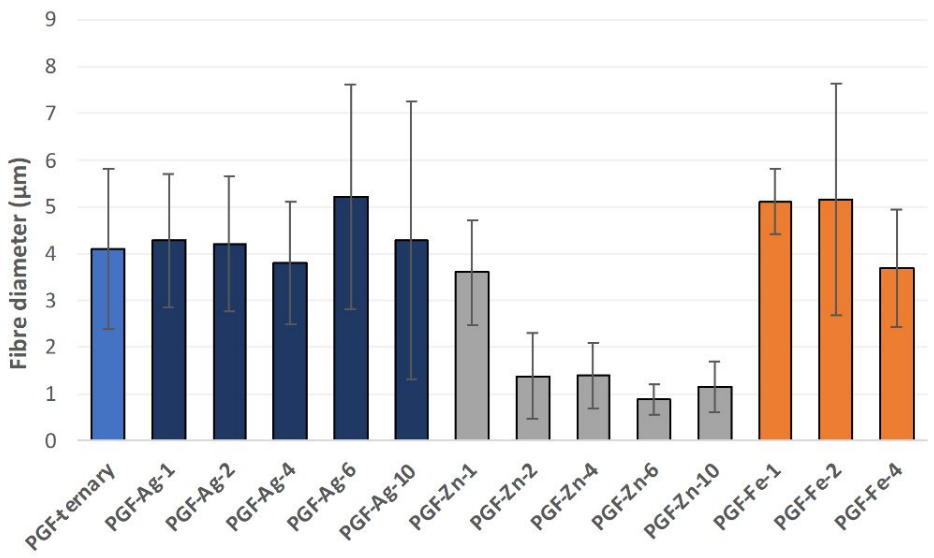

Figure S4. Mean fibre diameter of PGF-ternary, PGF-Ag, PGF-Zn and PGF-Fe, containing 1, 2, 4, 6 and 10 mol % of TMIs.
